# Supplementary material for: Single-molecule conductance of a chemically modified, π-extended tetrathiafulvalene and its charge-transfer complex with F4TCNQ
Source: Beilstein J Org Chem. 2015 Jun 24;11:1068–78. doi: 10.3762/bjoc.11.120 (PMC4505095; doi:10.3762/bjoc.11.120)
Supplement: File 1 — Detailed experimental procedures for the synthesis and characterization of 5, break junction experiments and theoretical methods. [file Beilstein_J_Org_Chem-11-1068-s001.pdf]

Supporting Information
for
Single-molecule conductance of a chemically modified π-extended tetrathiafulvalene and its charge-transfer complex with F_4TCNQ

Raúl García[1], M. Ángeles Herranz[1], Edmund Leary[*2], M. Teresa González[2], Gabino Rubio Bollinger[3], Marius Bürkle[4], Linda A. Zotti[5], Yoshihiro Asai[4], Fabian Pauly[6], Juan Carlos Cuevas[5], Nicolás Agraït[2,3] and Nazario Martín[*1,2]

Address: [1]Departamento de Química Orgánica, Facultad de Química, Universidad Complutense, E-28040 Madrid, Spain, [2]Fundación IMDEA Nanoscience, Campus de Cantoblanco, Universidad Autónoma, E-28048 Madrid, Spain, [3]Departamento de Física de la Materia Condensada, and Instituto "Nicolás Cabrera", Universidad Autońoma de Madrid, E-28049 Madrid, Spain, [4]Nanosystem Research Institute, National Institute of Advanced Industrial Science and Technology (AIST), Tsukuba, Ibaraki 305-8568, Japan, [5]Departamento de Física Teórica de la Materia Condensada, Universidad Autónoma de Madrid, Spain and [6]Department of Physics, University of Konstanz, D-78457 Konstanz, Germany.

Email: Nazario Martín[*] - nazmar@quim.ucm.es; Edmund Leary[*] - edmund.leary@imdea.org

*Corresponding author

Detailed experimental procedures for the synthesis and characterization of 5, break junction experiments and theoretical methods

1. General methods

Reagents for synthesis were used as purchased. Argon was used as inert gas when necessary. Column chromatography was carried out using silica gel (40–63 μm) from Fluka. Analytical thin layer chromatography (TLC) was done using aluminium sheets (20 x 20 cm) precoated with silica gel RP-18W 60 F254 from Merck, or aluminium foils (20 x 20 cm) covered with nano-silica gel from Fluka. UV active compounds were detected with a UV lamp from CAMAG at wavelengths λ = 254 or 366 nm.

NMR spectra were recorded on a Bruker Avance 300 spectrometer at 298 K using partially deuterated solvents as internal standards. Coupling constants (J) are denoted in Hz and chemical shifts (δ) in ppm. Multiplicities are denoted as follows: s = singlet, d = doublet, t = triplet, m = multiplet, br = broad. Matrix-assisted laser desorption ionization (coupled to a time-of-flight analyzer) experiments (MALDI-TOF) were recorded on a Bruker REFLEX III spectrometer. Infrared spectra were recorded in a Bruker Tensor 27 spectrophotometer with an ATR device. Absorption spectra were recorded on a Shimadzu UV–vis–NIR 3200 spectrophotometer. Electrochemical measurements were carried out on an Autolab PGSTAT30 potentiostat–galvanostat equipped with an electrochemical analysis software for windows version 4.8, in a 3-electrode single compartment cell. A glassy carbon electrode (GCE) was used as working electrode; as the counter electrode a platinum wire was used; the reference electrode was a solution of Ag/Ag^+ in acetonitrile. $TBAPF_6$ (0.1 M in THF) was employed as supporting electrolyte.

Compounds **1**–**3** [1], and **4** [2] were prepared following the reported procedures and showed the same characterization properties as described therein.

2. Synthesis and characterization of compounds 5

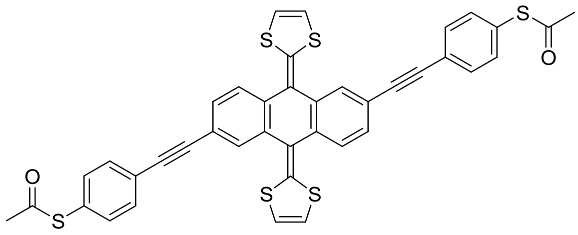

To a solution of bis(ethynyl)exTTF **3** (260 mg, 0.607 mmol) in anhydrous THF (120 mL) and under argon atmosphere, where subsequently added 1-acetylthio-4-iodobenzene (**4**, 424 mg, 1.53 mmol), bis(triphenylphosphine)palladium(II) chloride (42 mg, 0.06 mmol), copper iodide (11 mg, 0.06 mmol) and *N,N*-diisopropylamine (0.85 mL). The reaction mixture was then left stirring for 12 h after which the solvent was removed under vacuum. The isolated product was purified by column chromatography (hexane/dichloromethane 8:2) to afford a yellowish orange solid in 35% yield.

FTIR (KBr, cm^{-1}), ν: 3068, 2925, 2857, 2197, 1724, 1546, 1501, 1461, 1271, 1121, 1080, 829, 770.

^{1}H-NMR ($CDCl_3$, 300 MHz), δ: 7.85 (d, 2H, J = 1.5 Hz), 7.70 (d, 2H, J = 8.1 Hz), 7.59 (d, 4H, J = 8.4 Hz), 7.47 (dd, 2H, J_1 = 8.1 Hz, J_2 = 1.5 Hz), 7.42 (d, 4H, J = 8.4 Hz), 6.37 (s, 4H), 2.45 (s, 6H).

^{13}C-NMR ($CDCl_3$, 75 MHz), δ: 192.4, 137.0, 134.4, 133.2, 131.2, 128.3, 127.0, 126.9, 124.0, 123.6, 119.6, 119.4, 116.4, 90.4, 88.0, 28.7.

UV-vis (CH_2Cl_2) λ_{max} (ε): 391 (12272), 457 (13409), 563 (5909).

HRMS (MALDI-TOF) *m/z*: estimated for $C_{40}H_{24}O_2S_6$ = 728.0101; found = 728.0102.

3. Break junction experiments

A home-built scanning tunneling microscope (STM) as described previously [3] was used. As substrates commercially available gold samples on quartz (Arrandee), and as tip a freshly cut gold wire was used. For all measurements, a bias voltage (V), of 200 mV was applied between the tip and the substrate. A linear current-to-voltage converter with two amplification stages was used to measure the current (I) in the circuit. For the measurements presented in Figure 3 and Figure 4 the experiments were performed in air, after immersing the substrate in 10^{-4} to 10^{-3} M solutions in 1,2,4-trichlorobenzene for 30 min followed by drying under a flow of N_2. The gains used were 5.6 x 10^5 V/A and 1.2 x 10^8 V/A, after the first and second stages, respectively. For the experiments shown in Figure 5 and Figure 6, the substrate was immersed in 10^{-4} M solutions in CH_2Cl_2 for 15 min followed by drying under a flow of N_2. A protection resistor of either 100 kΩ (Figure 6) or 200 kΩ (Figure 5) was placed in series with the substrate–tip circuit. Our gains in this work were 10^7 V/A and 2 x 10^9 V/A (Figure 6) and 4.3 x 10^7 V/A and 8.6 x 10^9 V/A (Figure 5), after the first and second stages, respectively.

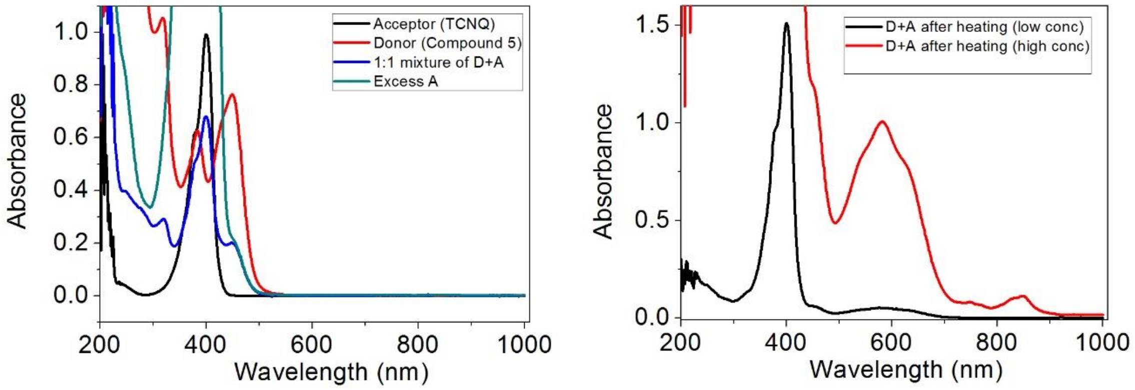

Figure S1: Left, UV–vis spectra of the neutral donor **5** (red), acceptor TCNQ (black), CT complex in acetonitrile (blue) and after adding a large excess of TCNQ (5 equiv) (green). Right, 1:5 mixtures of **5** and TCNQ after heating and at different concentrations.

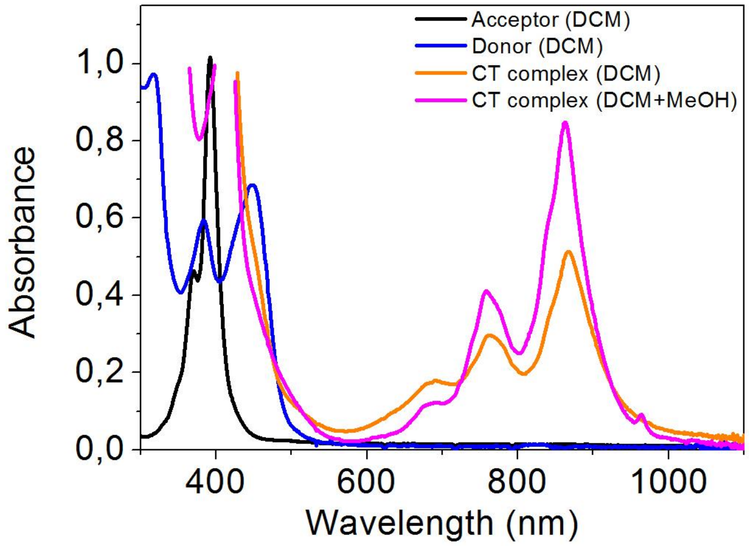

Figure S2. UV–vis spectra of the neutral donor **5** (blue), acceptor F_4TCNQ (black), CT complex in dichloromethane (orange) and after adding a few drops of methanol (pink).

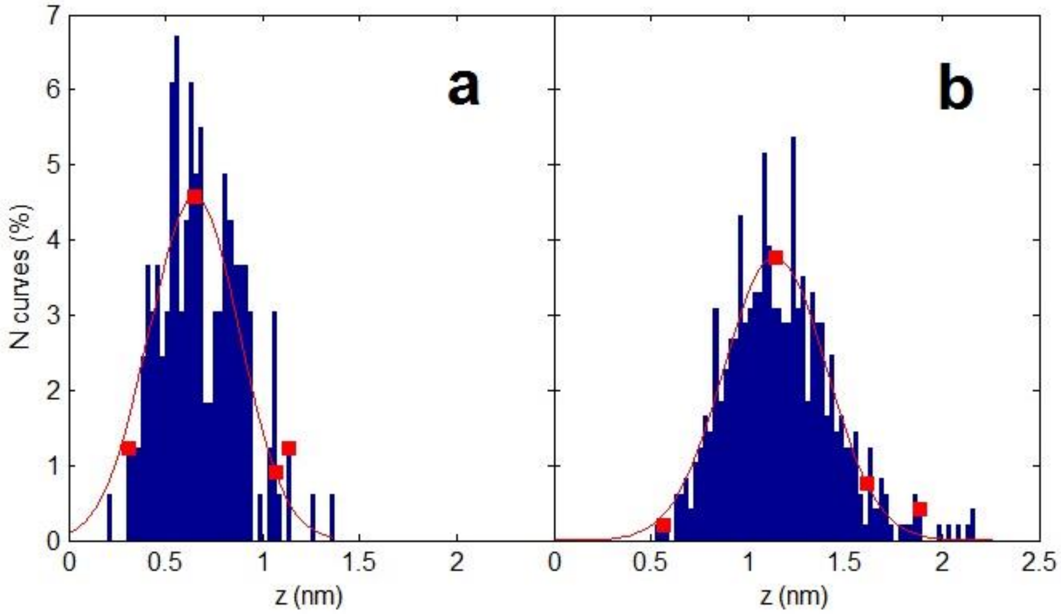

Figure S3: Histograms of the plateau lengths measured in each Gz trace determined to contain a plateau at the high (a) or low (b) conductance range, for the CT complex formed by compound 5 with F4TCNQ. The plateau length of each trace is calculated as the total displacement Δ*z* needed to change the junction conductance from 0.5 to 1 x 10^{-4} G_0 for the high plateaus (a) and 1 x 10^{-6} G_0 for the low plateaus (b).

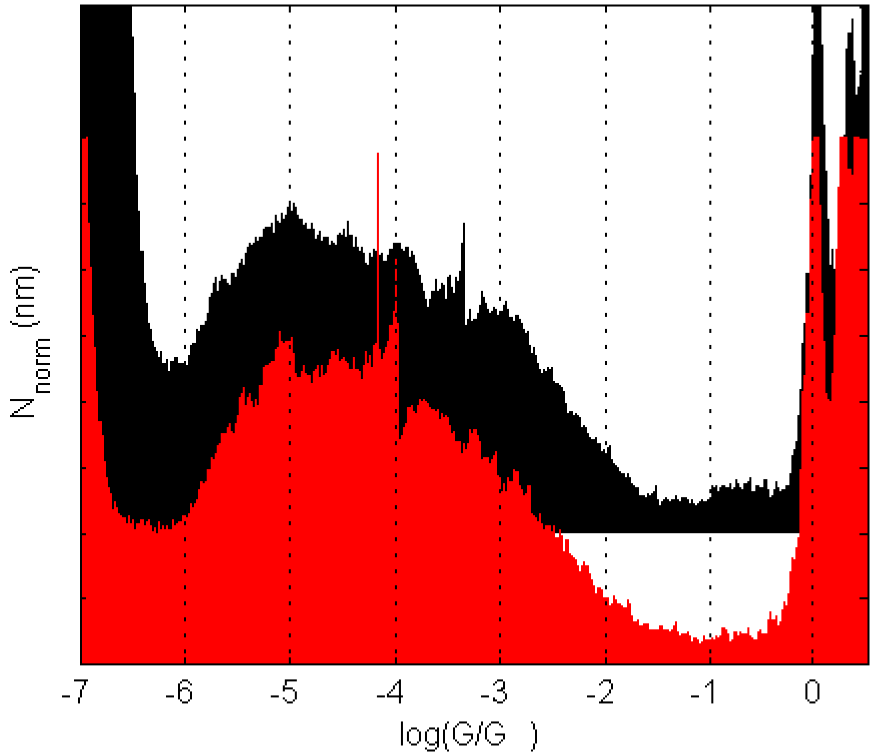

Figure S4: 1D conductance histograms of separate experimental runs (freshly prepared CT complex, new STM electrodes). In both cases we show the histograms of the separated conductance plateaus (all plateaus). Note that two different gains were used changing the lower limit in visible conductance and the part in the histogram we join the two channels. The general shape is clearly reproduced in both measurements.

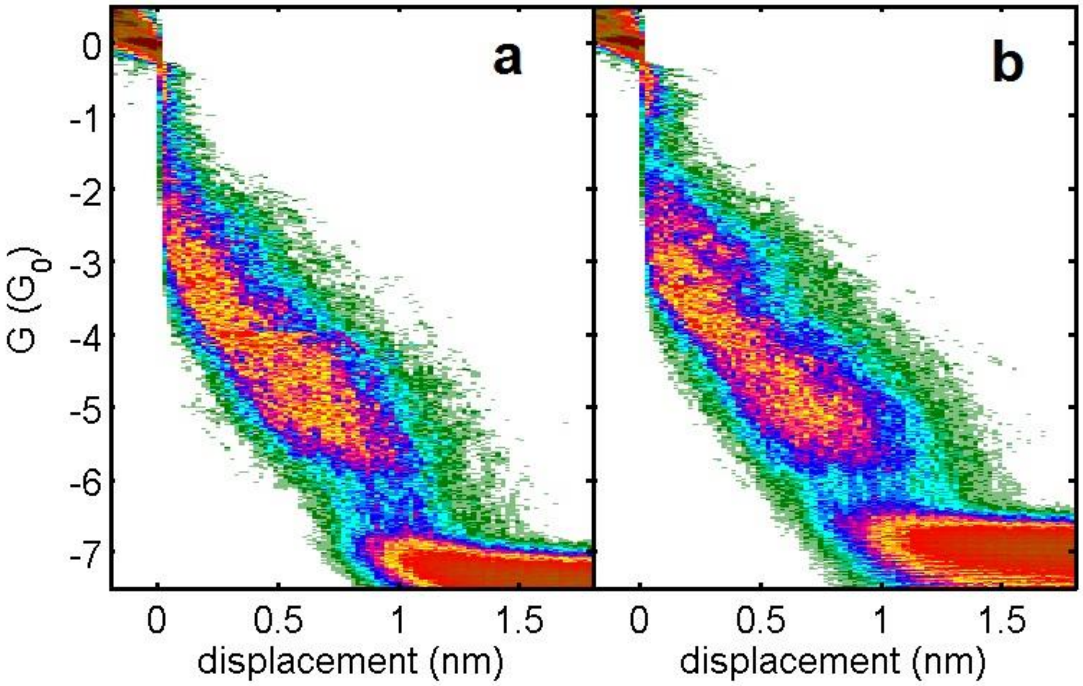

Figure S5: The 2D histograms of the same data as in Figure S3. We notice in experimental run (a) a less prominent feature close to 10^{-3} G_0 than in (b). The low conductance group, however, appears very similar in the two runs. This strengthens the idea that the low group corresponds to transport across the CT complex from thiol to thiol. In both runs the percentage of plateaus in the low conductance group was 10% (the total number of plateaus are: run a = 637/6443 (10%); run b = 485/4868 (10%).

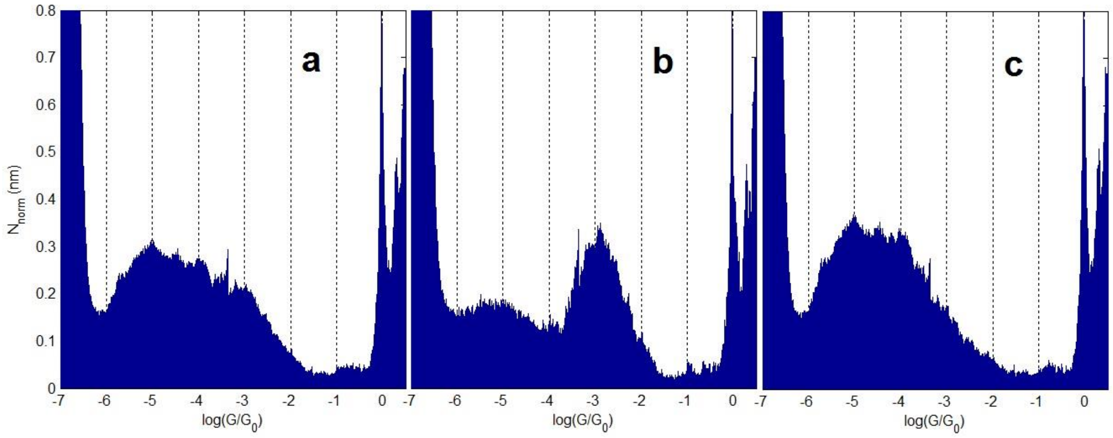

Figure S6: (a). 1D histogram of all conductance plateaus for the data presented in the main text (Figure 6). (b). Conductance histogram of the separated high plateaus. (c). Conductance histogram of the separated low plateaus. The plateaus were divided at a conductance of 1.6 x 10^{-4} G_0 ($\log(G/G_0) = -3.8$).

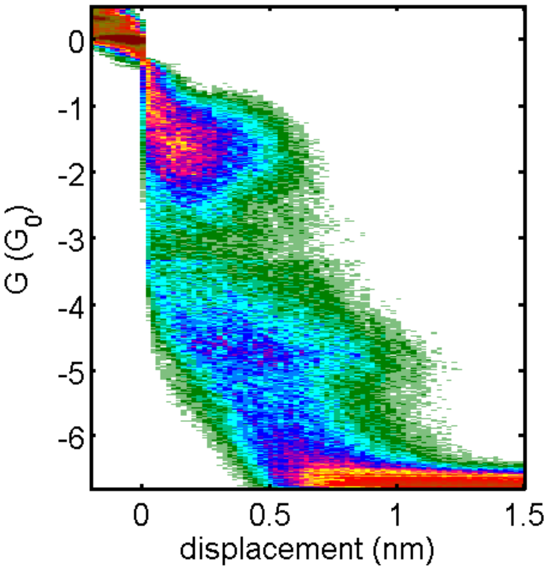

Figure S7: 2D histogram of a sample prepared only depositing the acceptor F_4TCNQ.

4. Theoretical methods

We employed the DFT-based transport method described in detail in reference [4], which is built on the quantum chemistry code TURBOMOLE 6.1 [5]. In all our calculations we used the BP86 exchange-correlation functional [6] and a split-valence basis set with polarization for all non-hydrogen atoms def-SVP [7]. In order to construct the junction geometries, we first relaxed the molecules in the gas phase. Then, we placed the relaxed molecules between two finite clusters of 20 (or 19) gold atoms and performed a new geometry optimization in which the molecule and the four (or three) outermost gold atoms on each side were relaxed, while the other gold atoms were kept frozen. The total energies were converged to a precision of better than 10^{-6} atomic units, and structure optimizations were carried out until the maximum norm of the Cartesian gradient fell below 10^{-4} atomic units. Subsequently, the size of the gold clusters was extended to about 120 atoms on each side in order to describe the metal–molecule charge transfer and the energy level alignment correctly. The information obtained on the electronic structure of the junctions within DFT was then transformed into the different transport properties by using non equilibrium Green's function techniques, as described in detail in reference [4]. To overcome the well-known problems related with the HOMO–LUMO gap underestimation in DFT, we applied the DFT + Sigma method [8] as described in detail in reference [9].

References

1. Illescas, B. M.; Santos, J.; Díaz, M. C.; Martín, N.; Atienza, C. M.; Guldi, D. M. *Eur. J. Org. Chem.,* **2007**, 5027–5037.

2. Pearson, D. L.; Tour, J. M. *J. Org. Chem.*, **1997**, *62*, 1376–1387.
3. González, M. T.; Leary, E.; García, R.; Verma, P.; Herranz, M. A.; Rubio-Bollinger, G.; Martín,N.; Agrait. N. *J. Phys. Chem. C,* **2011**, *115*, 17973–17978.
4. Pauly, F.; Viljas, J. K.; Huniar, U.; Haefner, M.; Wohlthat, S.; Buerkle, M.; Cuevas, J. C.; Schoen. G. *New J. Phys.,* **2008**, *10*, 125019.
5. Ahlrichs, R.; Bär, M.; Häser, M.; Horn, H.; Kölmel, C. *Chem. Phys. Lett.,* **1989**, *162*, 165–169.
6. Perdew, J. P. *Phys Rev B,* **1986**, *33*, 8822–8824.
7. Schäfer, A.; Horn, H.; Ahlrichs. R. *J. Chem. Phys.,* **1992**, *97*, 2571–2577.
8. Quek, S. Y.; Venkataraman, L.; Choi, H. J.; Louie, S. G.; Hybertsen, M. S.; Neaton, J. B. *Nano Lett.*, **2007**, *7*, 3477–3482.
9. Zotti, L. A.; Bürkle, M.; Pauly, F.; Lee, W.; Kim, K.; Jeong, W.; Asai, Y.; Reddy, P.; Cuevas, J. C. *New J. Phys.,* **2014**, *16*, 015004.
